# Management of Treatment‐Resistant Dermatitis Herpetiformis With Tofacitinib: A Case Report

**DOI:** 10.1002/ccr3.71410

**Published:** 2025-11-12

**Authors:** Armaghan Kazeminejad, Fatemeh Montazer, Mohammad Malekan

**Affiliations:** ^1^ Department of Dermatology, Antimicrobial Resistance Research Center, Communicable Diseases Institute Mazandaran University of Medical Sciences Sari Iran; ^2^ Firoozabadi Clinical Research Developement Unit (FACRDU), School of Medicine Iran University of Medical Sciences (IUMS) Tehran Iran; ^3^ School of Medicine Mazandaran University of Medical Sciences Sari Iran

**Keywords:** case report, dermatitis herpetiformis, Duhring's disease, JAK inhibitors, targeted therapy, tofacitinib

## Abstract

Tofacitinib may offer a safe and effective treatment alternative for patients with dermatitis herpetiformis unresponsive to dapsone and a gluten‐free diet, highlighting the potential role of JAK inhibition in refractory autoimmune skin diseases.

## 1 Introduction

Dermatitis herpetiformis (DH) is a rare, relapsing, and pruritic autoimmune dermatosis closely associated with gluten sensitivity. It is considered a specific cutaneous manifestation of celiac disease [1]. The pathogenesis involves IgA deposition in the dermal papillae, leading to neutrophilic inflammation and blister formation. Lesions typically appear on the extensor surfaces, including elbows, knees, and buttocks. While most cases respond to a gluten‐free diet and dapsone, a subset of patients develops treatment‐resistant disease, posing significant therapeutic challenges [2].

Recently, Janus kinase (JAK) inhibitors such as tofacitinib have emerged as potential therapeutic options for immune‐mediated skin disorders due to their role in modulating inflammatory pathways. However, there is limited evidence to guide the treatment of dapsone‐resistant DH, and only a few case reports have explored novel immunomodulatory agents, such as JAK inhibitors [3]. Here, we report a case of treatment‐resistant DH successfully managed with tofacitinib, a JAK1 and JAK3 inhibitor, highlighting its potential as a therapeutic option.

## 2 History and Examination

**FIGURE 1:**
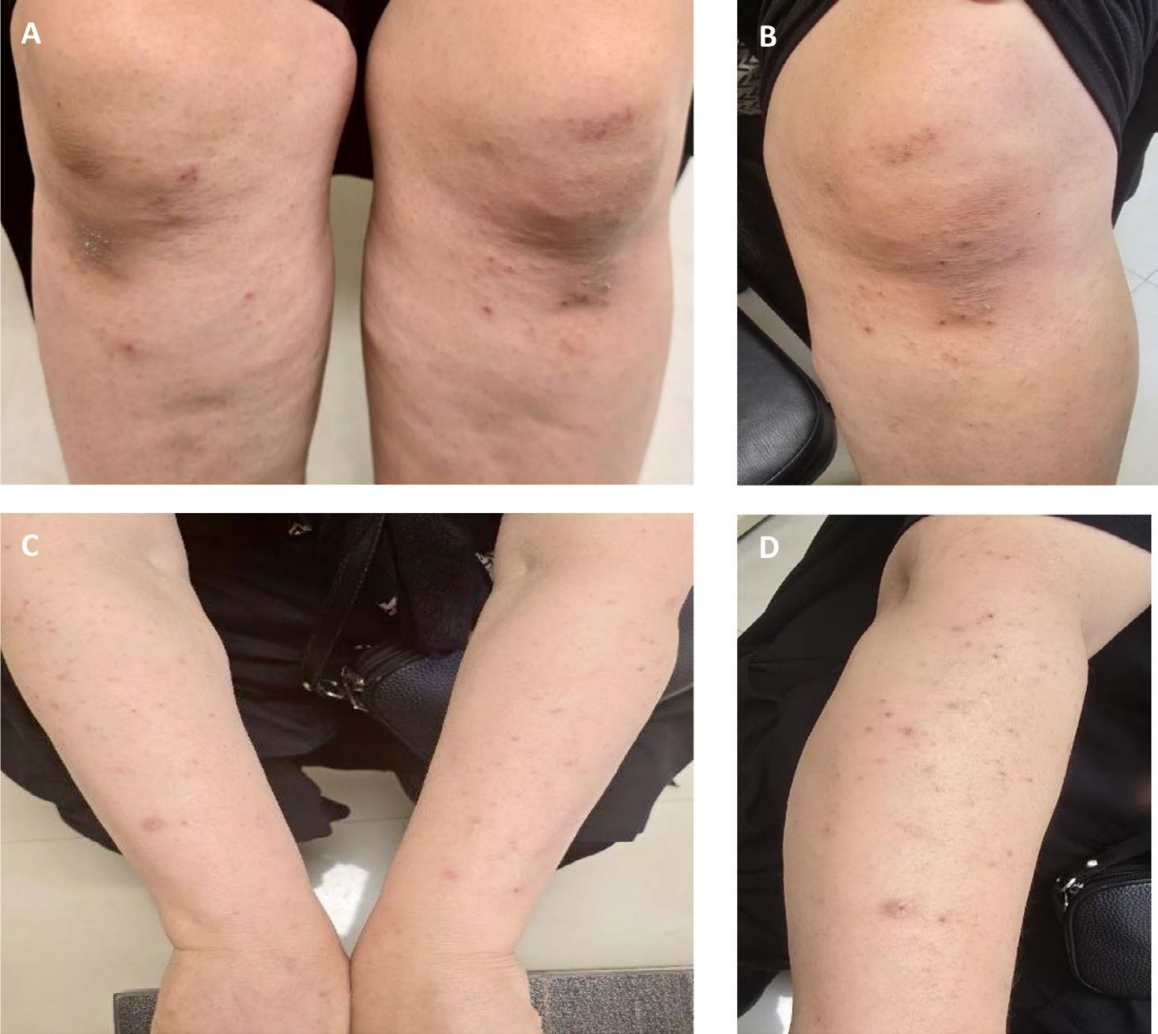
(A, B) Multiple erythematous papules, excoriations, vesicular lesions, and post‐inflammatory hyperpigmentation on the extensor surfaces of the knees; (C, D) Erythematous papules, excoriations, and post‐inflammatory pigmentation on the extensor surfaces of the forearms and elbows.

A 49‐year‐old woman presented with a two‐year history of intense pruritus on the extensor surfaces of her arms and legs. The itching significantly impaired her quality of life. Previous treatments included multiple courses of topical corticosteroids, which provided minimal relief. Her past medical history included celiac disease and mild seasonal allergies, managed with over‐the‐counter antihistamines, and no history of autoimmune diseases, or other dermatologic conditions. She reported no relevant family history and was not taking any regular medications. Physical examination revealed excoriated erythematous papules and vesicles on the extensor surfaces of the elbows and knees (Figure 1).

## 3 Differential Diagnosis, Investigations and Treatment

**FIGURE 2:**
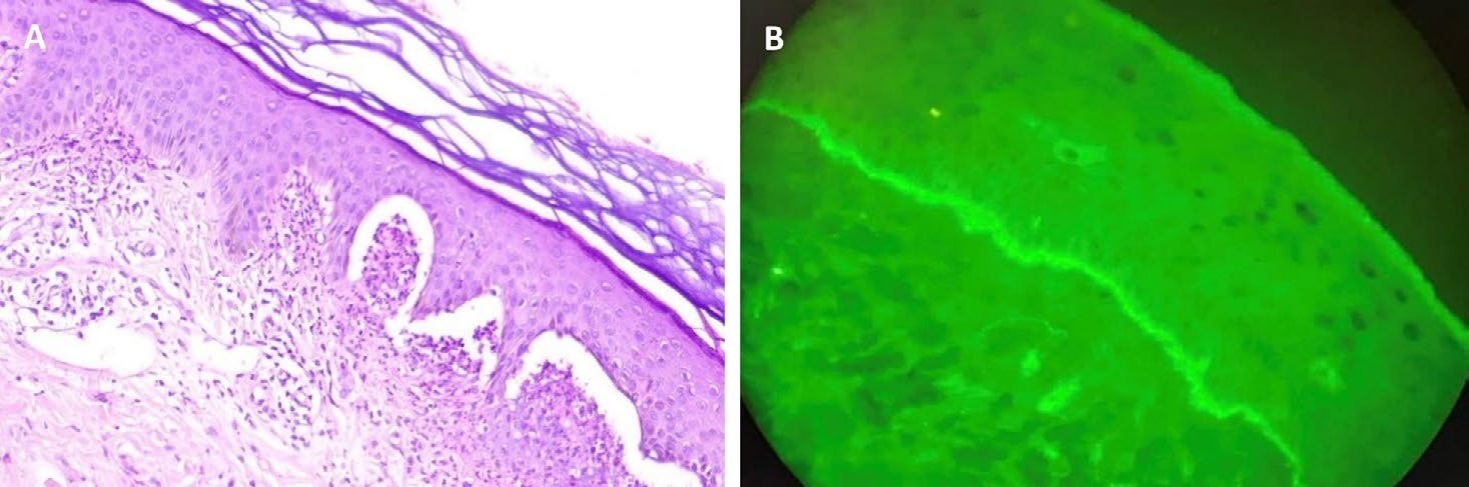
(A) H&E staining (×200) shows a mildly acanthotic epidermis with subepidermal vesiculations containing neutrophils and dermal papillary neutrophilic microabscesses. The papillary dermis exhibits mild perivascular and interstitial infiltration of neutrophils, lymphocytes, and eosinophils, with associated edema and ectatic vessels; (B) Direct immunofluorescence for IgA (×200) reveals granular IgA deposits along the basement membrane zone, consistent with dermatitis herpetiformis.

Several differential diagnoses were considered. These included linear IgA bullous dermatosis, bullous pemphigoid, eczema (particularly nummular or atopic dermatitis), and scabies. A skin biopsy was performed, and two independent histopathological reviews confirmed DH, demonstrating a mildly acanthotic epidermis with multiple subepidermal vesiculations containing neutrophils, as well as dermal papillary neutrophilic microabscesses. The papillary dermis showed mild perivascular and interstitial infiltration of neutrophils, lymphocytes, and eosinophils, along with edema and ectatic blood vessels. Direct immunofluorescence for IgA revealed granular deposits along the basement membrane zone, consistent with dermatitis herpetiformis (Figure 2).

Despite the initiation of standard treatment with oral dapsone, starting at 50 mg and increased to 100 mg daily, along with adherence to a strict gluten‐free diet, the patient reported no significant improvement in symptoms. Given the lack of response to conventional therapy, an off‐label trial of tofacitinib at a dose of 10 mg daily was initiated.

## 4 Conclusion and Results

After 1 month, the patient reported an 80% reduction in pruritus, with decreased erythema and fewer new lesions. No adverse effects were reported, and laboratory monitoring (complete blood count, liver, and renal function) remained normal. At the 3‐month follow‐up, the patient maintained significant symptom relief.

## 5 Discussion

JAK inhibitors are small‐molecule agents that modulate the JAK–STAT pathway, a key regulator of immune and inflammatory responses via cytokine signaling. Tofacitinib, which primarily inhibits JAK1 and JAK3 with partial JAK2 inhibition, is approved for conditions like rheumatoid arthritis (RA) and ulcerative colitis (UC), but has emerging applications in dermatologic diseases. By interrupting JAK‐mediated phosphorylation of STAT proteins, tofacitinib suppresses pro‐inflammatory cytokines such as interleukin (IL)‐17, IL‐23, and interferon‐gamma (IFN‐γ), which drive immune‐mediated tissue damage [4].

The pathogenesis of DH, a cutaneous manifestation of celiac disease, involves IgA deposition in the dermal papillae, triggering neutrophilic infiltration and intense pruritus, likely driven by gluten sensitivity and immune dysregulation [2]. The JAK–STAT pathway may contribute to DH by amplifying cytokine signaling, particularly IL‐17 and IL‐23, which promote neutrophil recruitment and epidermal inflammation [4]. Our patient responded to tofacitinib (10 mg daily) suggesting that JAK–STAT inhibition disrupts the inflammatory cascade in DH. This response aligns with the histopathological findings of neutrophilic clusters and eosinophilic infiltration, indicating that tofacitinib likely mitigated cytokine‐driven immune activation, offering a targeted approach to controlling refractory DH symptoms.

Similar case reported by Kahn et al. supports the efficacy of tofacitinib in DH management. Their study described a patient with DH who achieved significant symptom improvement with tofacitinib [5]. Both cases highlight the potential of tofacitinib as an alternative therapy for DH patients unresponsive to standard treatments, particularly when neutrophilic and eosinophilic inflammation predominates. While the precise mechanisms linking JAK–STAT signaling to DH require further investigation, these clinical outcomes suggest that targeting this pathway can effectively address the inflammatory components of DH.

In this case, serological testing for IgA antibodies to epidermal transglutaminase (TG3) and tissue transglutaminase (TG2), which can correlate with disease activity in DH, was not performed due to the patient's socioeconomic limitations. Nevertheless, the diagnosis was confidently established based on the clinical presentation, histopathological findings, and direct immunofluorescence demonstrating granular IgA deposition, which is considered sufficient for confirmation.

This case highlights tofacitinib as an effective treatment for refractory DH, achieving significant symptom relief where dapsone and a gluten‐free diet proved inadequate. Rapid improvement was sustained at 3 months without adverse effects, suggesting a critical role for JAK–STAT signaling in DH pathogenesis.

## Author Contributions

**Armaghan Kazeminejad:** project administration, validation, writing – original draft, writing – review and editing. **Fatemeh Montazer:** data curation, investigation, validation, visualization. **Mohammad Malekan:** conceptualization, supervision, writing – original draft.

## Consent

Written informed consent was obtained from the patient for publication of this case report and any accompanying images.

## Conflicts of Interest

The authors declare no conflicts of interest.

## Data Availability

Data will be available upon reasonable request from the corresponding author.
